# Classification Based on Extraction Socket Buccal Bone Morphology and Related Treatment Decision Tree

**DOI:** 10.3390/ma15030733

**Published:** 2022-01-19

**Authors:** Larissa Steigmann, Riccardo Di Gianfilippo, Marius Steigmann, Hom-Lay Wang

**Affiliations:** 1 Department of Periodontics and Oral Medicine, School of Dentistry, University of Michigan, Ann Arbor, MI 48109, USA; lasteigm@umich.edu (L.S.); rdgianfi@umich.edu (R.D.G.); 2 Steigmann Institute, 69151 Neckargemünd, Germany; drsteigmann@gmail.com

**Keywords:** alveolar ridge preservation, tooth socket, tooth extraction, alveolar ridge augmentation, endosseous dental implantation

## Abstract

Background: Alveolar ridge preservation (ARP) can successfully reduce volumetric ridge changes. However, there is still no consensus on what technique is the most advantageous for each specific clinical scenario. Hence, the aim of the present paper was to provide a treatment decision tree to guide the choice of predictable ARP procedures based on extraction socket buccal bone morphology and integrity. Material and Methods: Three socket types (ST) are proposed and discussed based on buccal bone morphology (intact, dehiscence or fenestration). Results: A decision tree for ARP was developed in order to merge ST classification with suitable treatment modalities. In the decision tree, the issue of when to allow unassisted healing or ARP was discussed. Described methods included bone grafting and collagen plug, and absorbable membrane or non-resorbable membrane, with or without flap elevation. Conclusion: A decision tree for ARP procedures was provided to guide clinicians towards the most conservative and predictable treatment approach based on remaining socket anatomical structures after extraction.

## 1. Introduction

Tooth extraction results in significant ridge resorption, with alveolar bone and soft-tissue changes that may jeopardize esthetics and complicate functional rehabilitation [1,2]. Alveolar ridge preservation (ARP) therapies have been found to be effective in reducing volumetric changes without completely preventing the process of resorption [3,4,5,6]. The beneficial effect of ridge-preservation interventions was recently updated in the XV European Workshop in Periodontology [7], which quantified an overall 1 to 2.5 mm reduction in ridge resorption for ARP compared to unassisted healing with more dramatic differences in the coronal buccal aspect of the ridge.

A plethora of materials and surgical techniques has been described, evaluated and compared in well-designed randomized clinical trials (RCT) [8,9,10,11]. However, despite intensive investigations and previously proposed classifications [12,13,14], there is still no consensus on what technique is the most predictable. A possible explanation could derive from the high anatomical variability of the extraction sockets that would affect the regenerative potential of the many proposed techniques with different magnitudes. Recently, one study reported that thin buccal bone had more extensive bone volume resorption [8], while thick bones made the effect of ARP negligible [15,16]. As described, buccal bone integrity including thickness was one of the strongest predictors of decreased volume changes irrespective of other phenotypic variables. Available literature has focused primarily on the type of grafting material, membrane selection and case selection for immediate implant placement [13,14], but less is known on the decision of how and when to apply these materials [17]. In the era of personalized medicine, the choice among available protocols should be adapted according to site-related bone morphometrics at the time of treatment.

Therefore, the aim of this study was to provide a decision tree that would guide clinicians towards the most suitable ARP technique, excluding primary wound-closure approaches and immediate implant-placement approaches, depending on buccal bone anatomical circumstances of the extraction socket.

## 2. Materials and Methods

An extensive literature review was conducted on notable journals of Periodontics, Implantology and Oral Surgery to investigate the most relevant phenotypic features of the extraction socket that could impact the outcome of ARP. Buccal bone thickness, integrity, dehiscence or fenestration, which have been found to affect outcomes after ARP [8,15,16,18,19,20,21], were used as discriminants of the proposed classification and decision tree. Our proposed “socket type” (ST) classification divided the socket into 3 according to main categories (Table 1):

- Socket type 1 (ST1) described an extraction socket with intact buccal bone;
  - ST1 Subclass A (ST1A): buccal bone thickness of ≥1.0 mm;
  - ST1 Subclass B (ST1B): buccal bone thickness of <1.0 mm;
- Socket type 2 (ST2) referred to sockets with buccal bone fenestration;
- Socket type 3 (ST3) was used for buccal bone dehiscences that would compromise bone height. It was further subdivided according to severity and extent of the buccal dehiscence;
  - ST3 Subclass A (ST3A) was used when a dehiscence is present and extending less than 1/3 of the length of the buccal bone wall;
  - ST3 Subclass B (ST3B) was used when a buccal dehiscence is present and extending 1/3 to 2/3 of the total length of the alveolus;
  - ST3 Subclass C (ST3C) was used for severe dehiscence passing more than 2/3 of the facial bone of the alveolus.

With knowledge of socket-type classification, a decision tree was proposed to match each anatomical scenario to the most predictable treatment modality excluding primary wound-closure approaches and immediate implant-placement approaches.

## 3. Results

**Figure 1:**
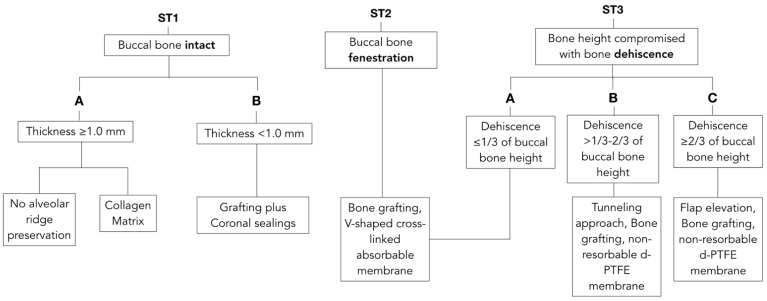
Decision tree matching anatomical features with suggested treatment options. Unassisted healing and most documented techniques for alveolar ridge preservation have been related with the ST Classification. Each clinical scenario is correlated with the most suitable treatment approach to allow maximum regenerative potentials with the most conservative intervention.

**Figure 2:**
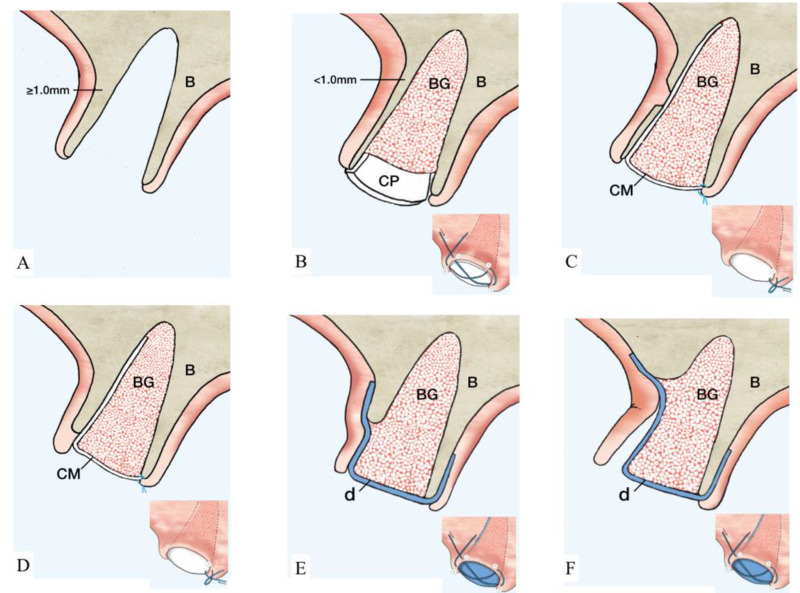
Illustration of ST-Classification with suggested treatment options. (**A**): ST1A. Extraction socket with thick (≥1 mm) and intact buccal bone. No further treatment is needed. Optionally, a collagen matrix may be placed to facilitate homeostasis. B showed the management of ST1B socket. (**B**): Thin (<1 mm) and intact buccal bone. Suggested treatment option is a particulate grafting material with collagen dressing on the coronal aspect. The collagen plug is secured by a cross suture as illustrated. (**C**): Buccal bone with a bony fenestration regardless of buccal bone thickness. An absorbable membrane (marked in white) is placed inside the socket and, subsequently, particulate grafting material is added. The membrane is sutured to the palatal tissue using single interrupted suture. Treatment for ST3 sockets is reported in D–F. (**D**): The length of the dehiscence is ≤1/3 of buccal bone height. The recommended therapeutic approach coincides with the description of C. (**E**): Buccal dehiscence extends >1/3–2/3 of buccal bone height. At this point, the utilization of a non-resorbable d-PTFE membrane (marked in blue) is indicated. The membrane is positioned below the tunneled soft tissue and on top of the buccal bone covering the bone-grafting material. A cross suture is placed on top of the d-PTFE membrane. (**F**): Dehiscence surpasses ≥2/3 of buccal bone height. At this point, flap elevation is required and a d-PTFE membrane (marked in blue) is placed on top of the bone-grafting material and covered by a cross suture. The membrane is left in place for 4–6 weeks according to physiologic bone maturation. (Abbreviations. B: native bone. BG: particulate bone grafting. CP: collagen plug. CM: collagen membrane. d: dense polytetrafluoroethylene membrane.)

Prior to extraction interventions, the treatment plan should include a determination of buccal bone dimensions with its vertical and horizontal components, as well as the presence or absence of fenestrations or dehiscence. Preoperative cone-beam computed tomography (CBCT) for assessing socket anatomy is beneficial and often recommended. However, with the goals of providing clinical guidance for day-to-day practice and reducing radiation doses, bone integrity may be determined by using sulcular bone sounding with a probe. CBCT can be obtained for more accurate investigation of anatomy in challenging cases, or for immediate implant placement when needed. After tooth extraction, the alveolar socket must be assessed. Careful evaluation of buccal bone integrity is performed with the probe and palpation to determine the socket type according to the proposed classification. Decision tree and simplified drawing of proposed treatments are presented in Figure 1 and Figure 2, respectively.

**Figure 3:**
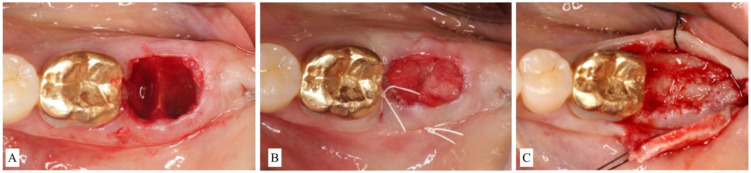
(**A**): Clinical view of alveolar socket after extraction of a second molar. The buccal bone was preserved in height but thinner than 1.0 mm. The case was classified as Socket type 1B (ST1B). (**B**): The socket was filled with particulate bone-grating material and coronally sealed with a collagen plug. (**C**): Surgical re-entry at the time of implant placement showed successful ridge preservation.

- Socket type 1 (ST1) is treated depending on the thickness of the buccal bone.
  - ST1A is treated with a collagen plug wound dressing matrix or by simple unassisted healing.
  - ST1B sockets are treated with a particulate allograft or xenograft placed inside the alveolus. Bone-grafting material is applied to fill the extraction socket 1–2 mm below the alveolar crest. The remaining coronal aspect is then sealed either by a bioabsorbable collagen plug wound dressing matrix [22,23] or by an autogenous soft-tissue graft especially in a highly esthetically demanding maxillary anterior region [24] (Figure 3A–C).

Collagen plug wound dressing matrices have been shown to contribute to coagulation and support homeostasis [25] and may, therefore, be added to initiate homeostasis. Thick buccal bone (≥1 mm) [16] makes the effect of ARP negligible, and no additional grafting is needed to retard dimensional changes. Unassisted healing offers advantages in terms of faster healing, higher percentage of vital bone and reduced cost for the patient.

**Figure 4:**
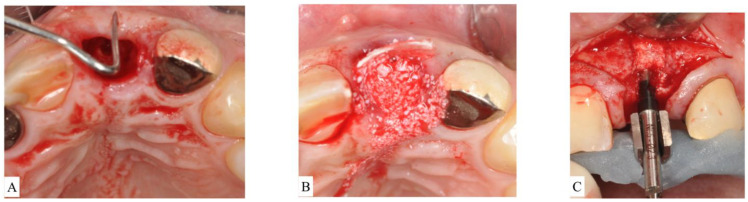
(**A**): After extraction of the upper left central incisor, socket anatomy displayed dehiscence of the facial bone. The socket was classified as Socket type 3A (ST3A). (**B**): A collagen cross-linked membrane was trimmed to have a V-shape and then placed over the dehiscence while still protruding out of the socket. Bone-grafting particulate was placed to fill the socket. The membrane was reflected to cover bone grafting, and sutures were placed to stabilize the membrane. (**C**): Surgical re-entry at the time of implant placement revealed successful ridge preservation and allowed desired implant positioning.

**Figure 5:**
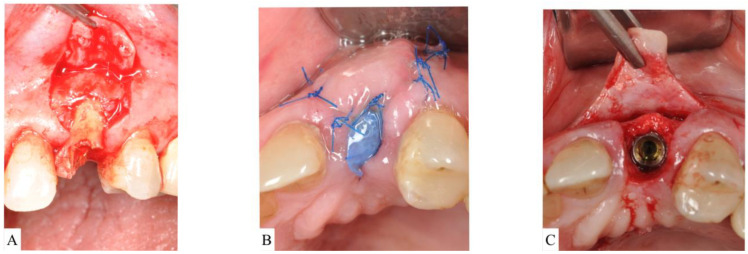
(**A**): Flap reflection apical to the upper left unrestorable maxillary canine showed visible dehiscence of the facial bone. The socket was classified as Socket type 3C (ST3C). (**B**): A d-PTFE membrane is placed to cover the particulate bone. The flap is sutured without attempting primary closure. (**C**): Surgical re-entry at the time of implant placement showed adequate bone volume for installation of an implant fixture in a restorable position.

For buccal bone thicknesses less than 1 mm, extraction often results in significant bone resorption and, thus, may compromise the future implant placement [16]. Hence, the socket is often treated with a mineralized bone plug technique [22] or similar approaches (bone graft then covered with collagen wound dressing material or soft-tissue graft):

- Socket type 2 (ST2) refers to socket anatomy characterized by buccal fenestration. This type of socket is treated with an ice-cream-cone approach [26], which uses a V-shaped collagen membrane placed inside the buccal socket wall without the need for flap elevation. The socket is then filled with particulate bone graft. The top part of the membrane is then moved palatally and secured with interrupted sutures. The membrane aims to protect the bone graft on the deficient area of fenestration regardless of buccal bone thickness.
- Socket type 3 (ST3) is treated with particulate bone-grafting material covered by absorbable or non-resorbable cell-occlusive membrane.
  - In ST3A, dehiscence is limited to the coronal half of the socket and the buccal bone is maintained in the apical portion. This classification can be treated the same manner as ST2. Briefly, an absorbable V-shaped collagen membrane is placed into the bone socket’s lining to cover bone grafting, as previously described for the ice-cream-cone technique (Figure 4A–C).

- ST3B is managed by a tunnel approach. A tunnelling instrument is used to separate the periosteum away from the bone, and bone grafting is applied. Then, a non-resorbable dense-polytetrafluoroethylene (d-PTFE) membrane [27] was tugged into the created space to cover the graft and at least 3 mm of native bone on the buccal and on the lingual/palatal side. Sutures were placed from the buccal to the lingual flap to secure the d-PTFE membrane. Tunneling is attempted first as a conservative alternative to flap elevation in this category.
- ST3C requires flap elevation on both buccal and lingual sides. Bone-grafting materials are then placed inside the socket and covered with a d-PTFE membrane. Bone grafting should be slightly overcontoured (2 mm) on the horizontal dimension to compensate for expected shrinkage (Figure 5A–C).

It is important to stress that collagen or d-PTFE membranes are intentionally left exposed and lack of contact with adjacent teeth is ensured. Indeed, no efforts for primary closure are pursued in any of the categories of the proposed decision tree.

Generally, the d-PTFE membrane is removed 4–6 weeks after placement to allow better vascularization and uneventful healing.

## 4. Discussion

Buccal plate integrity and its thickness are of significant predictive importance for the self-regenerative process of socket healing [8,16] and are used to guide the choice on whether ARP is needed. Thick intact buccal bone (≥1 mm) optimizes the spontaneous regenerative potential of the healing socket and can be left untreated (as suggested in the case of ST1A), while thin or compromised buccal bone (ST1B) poses a high risk of ridge collapse and requires intervention.

The choice of bone grafting depends on expected timing of implant surgery, clinician’s preference and material availability. Depending on their origin, bone-grafting materials have different properties that define their viability in bone formation [28]. Xenogenic bone graft is the material of choice for maintain long-term ridge dimensions [29], whereas allogenic bone graft provides biologic activity, greater bone metabolism and is potentially osteoinductive if demineralized [30]. The collagen matrix mechanically protects grafting material and facilitates clot formation, homeostasis and wound stabilization [31].

In ST1B cases, the grafted extraction socket may be sealed with a collagen plug wound dressing matrix or an autogenous soft-tissue punch [22,24,32]. The decision is primarily dictated by the extraction socket’s anatomy. The soft-tissue punch, described in the literature as socket-seal surgery [24], is primarily indicated in the maxillary anterior region. Autogenous soft tissue provides aesthetic advantages by increasing keratinized tissue, preventing immediate collapse of the ridges, and simplifying possible additional soft or hard tissue augmentation [33,34]. However, challenges related with the sloughing and scarring of autogenous soft tissue should be taken into consideration [35].

Discontinuities of buccal bone, known as fenestrations or dehiscence, significantly increase the risk of complications, especially in cases of advanced rehabilitation [36], and would be best treated with the aid of barrier membranes. Membranes aim to prevent soft-tissue invagination, allowing volume stabilization with cell-occlusive properties [37]. After membrane placement, local blood-vessel growth allows the recruitment of migratory mesenchymal stem cells (MSCs) to the surgical site and the membrane’s surface. These cells then proliferate and differentiate into mature osteoblasts responsible for bone matrix formation, which is essential during the regeneration process [38]. In the case of ST2 and ST3A, the use of a cross-linked absorbable membrane is suggested since it shows slower resorption rates compared to non-cross-linked collagen membranes [39]. The severity and extent of bone deficiency in ST3B requires a more rigid membrane to act as a bony wall in supporting inserted bone-grafting materials. Additionally, d-PTFE membranes allow volume stability and avoid ridge collapse while facilitating cellular adhesion to the membrane’s surface, shielding bacterial invasion and forming a hermetic seal [40,41]. Phenotypic architectural conservation of the soft tissue is retained while assisting underlying socket healing, resulting in beneficial clinical outcomes [27,42,43]. Notably, no primary closure is required when using a d-PFTE membrane in ST3B and ST3C [44]. Aiming for primary closure results in a shift of the mucogingival complex palatally. An intentionally non-submerged protocol results in greater preservation of keratinized tissue width and thickness [45]. These sustained soft-tissue dimensions will prevent possible soft-tissue recession and enhance aesthetical outcomes [46]. In the rare case where there is a simultaneous occurrence of fenestration and dehiscence, the severity and extent of dehiscence will dictate the appropriate treatment approach.

Early implant placement with simultaneous contour augmentation is suggested if the anatomy is so unfavorable as to discourage attempting any ARP techniques. According to this protocol, the extraction socket is left to heal untreated for 4 to 8 weeks. After soft-tissue closure but before significant changes in the bone contour, flaps are elevated, implants are placed and the bone contour is augmented with a simultaneous guided bone regeneration procedure [47]. Applying thick buccal bone facials to the implant facilitates improved peri-implant probing and lowers the risk for peri-implantitis [48,49]. The contour-augmentation approach showed long-term favorable results for implant survival and stability of the buccal bone contour [50].

We acknowledge the limitations of the current decision tree. The alveolar size and/or dimensions may impact the healing potential of the extraction socket with unassisted healing as well as with ARP procedures. However, in this decision tree, we focused on the thickness of the buccal bone and its indications for ARP. Furthermore, the anatomical region (maxilla vs. mandible/anterior vs. posterior) may provide different healing patterns. However, the proposed ARP decision tree should be the same regardless of the anatomical region since it is based upon the integrity as well as the thickness of the buccal plate.

Finally, the possibility of immediate implant placement was intentionally not included in the present decision tree. The decision regarding immediate implant placement follows different criteria, such as the amount of bone apical to the socket that is available for primary stability, the bucco-lingual position of the socket compared to ridge width and thickness, the height of the soft tissue, smile line and the patient’s esthetic concerns. These are in addition to the proposed phenotypic socket features for the ST classification.

## 5. Conclusions

A clinically based decision tree was proposed to provide a conservative and predictable alveolar ridge preservation treatment based on buccal bone anatomical structures after tooth extraction.

## Figures and Tables

**Table 1:** Socket type (ST) classification. Anatomy of the post-extractive socket has been classified in three categories based on buccal bone characteristics including height, thickness and presence of dehiscence or fenestration. ST classification represents an evaluation of the self-contentive anatomy of the socket that ranges from highest regenerative potential for ST1 to lowest for ST3C.

*CLASS*	ST1		ST2	ST3	
*DEFINITION*	**Buccal bone intact**		**Buccal bone fenestration**	**Buccal bone dehiscence** Dehiscence height:	
	**A**	Thickness ≥ 1 mm		**A**	≤1/3 of buccal bone height
	**B**	Thickness < 1 mm		**B**	1/3–2/3 of buccal bone height
				**C**	≥2/3 of buccal bone height

## Data Availability

Not available.
